# Universal Adhesives: Setting Characteristics and Reactivity with Dentin

**DOI:** 10.3390/ma12101720

**Published:** 2019-05-27

**Authors:** Dimitris Papadogiannis, Maria Dimitriadi, Maria Zafiropoulou, Maria-Dimitra Gaintantzopoulou, George Eliades

**Affiliations:** Department of Biomaterials, School of Dentistry, National and Kapodistrian University of Athens, 115 27 Athens, Greece; mar.dimitriadi82@gmail.com (M.D.); maria.zaf28@hotmail.com (M.Z.); dent4mar@yahoo.com (M.-D.G.); geliad@dent.uoa.gr (G.E.)

**Keywords:** universal adhesives, degree of cure, oxygen inhibition, hardness, interaction with dentin, bond strength

## Abstract

The aim of the study was to evaluate the performance of six commercially available universal dental adhesives: Adhese Universal (ADU), All-Bond Universal (ABU), Clearfil Universal Bond Quick (CBQ), G-Premio Bond (GPB), Prelude One (PRO) and Scotchbond Universal (SBU). The properties tested were: (a) degree of C=C conversion (DC%); (b) Vickers micro-hardness (VHN); (c) extent of oxygen inhibition (OI/μm), all related with the adhesive film properties; (d) extent of dentin demineralisation (DM%), insoluble salt formation (AS%); and (e) shear bond strength (SBS, self-etch mode) related to the adhesive-dentin interactions. Statistical analysis (α = 0.05) was performed by one-way ANOVA and Tukey’s test (DC%, VHN, OI, DM% AS%) and Weibull analysis (SBS, *σ*_0-_*β*). The DC ranged from 67.2–82.5% (all >GPB), OI from 5.6–18.6 μm (SBU > ADU, GPB, ABU > CBQ > PRO), microhardness from 1.1–6.6 VHN (SBU > ADU > ABU > CBQ > PRO > GPB: not measurable), DM from 69.3% (GPB) to 16–12.5% (CBQ, SBU, ADU) and 13.2–10.6% (ABU, ADU, PRO), in homogeneous groups and AS from 26–15.9% (ABU, CBQ > GPB, PRO, ADU, SBU). For SBS the *σ*_0_ (characteristic life) ranged from 29.3–16.6 MPa (CBQ, ADU, ABU, SBU > PRO > GPB), the *β* (reliability) from 5.1–9.7 (*p* > 0.05). All failure modes were of mixed type (adhesive and composite cohesive). Although all these adhesives were based on the 10-methacryloyloxydecyl dihydrogen phosphate (10-MDP) adhesive monomer, the different co-monomers, solvents and catalysts led to variations in their film properties, reactivity and bonding capacity with dentin.

## 1. Introduction

Despite significant improvements in adhesive systems, the bonded interface of dental hard tissues with resinous restoratives and luting agents remains the weakest area of tooth-coloured restorations [1,2]. Universal adhesives, the latest development in the field, maintaining the “all-in-one” philosophy [3], can be used as self-etch, etch-and-rinse or enamel selective-etch agents for bonding direct and indirect restorations to enamel and dentin [4,5]. Since the acidic monomers of these adhesives have already been introduced as primers for other substrates (i.e. alloys, polycrystalline ceramics), a multi-mode treatment has been instructed for these products. In some, a silane agent was incorporated for compatibility with glass-ceramic materials [4,5], thus providing a universal coupling agent.

The universal adhesives contain monomer blends of mild to moderate acidity (phosphate, carboxylic etc.) in reduced concentrations compared with their precursors [6], conventional dimethacrylate cross-linkers, non-acidic emulsifying monomers, catalysts for light- or dual-curing and a proper selection of solvents to enhance monomer spreading and substrate infiltration capacity. Most studies published on these adhesives are focused on bond strength and bond durability with dental hard tissues under different testing protocols [7,8,9]. It has been shown that when universal adhesives were bonded to enamel employing the self-etch mode, lower bond strength values were obtained in comparison with the etch-and-rinse mode. However, acid-etching of dentin did not improve the bonding efficiency of these materials [3,10]. Furthermore, collagen destabilization and enzymatic degradation, induced by the very low pH of conventional acid-etchants and the ionic demineralization mechanism, have raised questions on the long term efficacy of strong acidic treatments on dentin [11]. Currently, self-etch modalities using monomers with mild acidity and water-insoluble salt formation capacity with dentin, such as 10-methacryloyloxydecyl dihydrogen phosphate (10-MDP), have been considered as the most reliable treatment for dentin [12,13,14]. This is probably the main reason why most universal adhesives contain 10-MDP as their main adhesive monomer. On the other hand, little data is available for the universal adhesives regarding film forming properties and bonding mechanism [15,16,17], with most information provided by the manufacturers. Considering that these materials appeared as a new trend in dental adhesion due to the ongoing quest for clinical step reduction and improved technique sensitivity [18], further investigation of their mechanisms and properties is deemed important.

The aim of the present study was to comparatively evaluate the laboratory performance of commercially available universal adhesives with various film-forming properties, bonding mechanisms and interfacial strength with dentin. The null hypothesis was that there is no statistically significant difference between the universal adhesives tested in respect to their curing efficiency, extent of oxygen inhibition, hardness, reactivity and bond strength with dentin. 

## 2. Materials and Methods

The six adhesives tested and their compositions are listed in Table 1. The properties tested were: (a) degree of conversion; (b) the extent of oxygen inhibition; (c) hardness; (d) interactions with dentin; and (e) shear bond strength with dentin in the self-etch mode.

### 2.1. Degree of C=C Conversion (DC)

For this test, disk-shaped silicone rubber moulds (Ø = 7 mm, h = 1 mm) were bonded to microscopic glass-slides, a drop of each adhesive was placed in each mould and solvents were evaporated with a mild stream of warm air (40 °C) for 1 min. Additional adhesive was applied with the same procedure, until the moulds were overfilled. Another glass-slide with an attached mylar strip was placed onto the mould to level the free surface and to prevent oxygen inhibition. The specimens were light-cured for 15 s with an LED curing unit (Radii Plus, SDI, Bayswater, Victoria, Australia, 1.5 W/cm^2^ intensity at standard, high-intensity mode), with the tip of the curing unit in contact with the glass-slide and subsequently stored for 10 min (37 °C, dark/dry conditions). The top glass-slide was then removed and the directly irradiated surface was used to measure the DC% by attenuated total reflectance Fourier transform infrared (ATR-FTIR) spectroscopy. The central part of each specimen was slightly pressed against a class III diamond reflective element (1 × 1 mm^2^) of an attenuated total reflectance (ATR) cell (Golden Gate, Specac, Orpington, UK) with ZnSe focusing lenses, which was attached to a Fourier transform infrared (FTIR) spectrometer (Spectrum GX, Perkin-Elmer, Buckinghamshire, UK). Spectra were acquired under the following conditions: 2000–750 cm^−1^ wavenumber range, 4 cm^−1^ resolution, 40 scans co-addition, ~2 μm depth of analysis at 1000 cm^−1^. Spectra were also acquired from uncured materials after solvent evaporation, which served as a reference. The degree of conversion (DC%) of each specimen, defined as the amount of C=C bonds converted into C–C, was estimated on a relative percentage basis with the two-band method and the tangent baseline technique [19]. The peaks of the aliphatic C=C bond stretching (str) vibrations (1636 cm^−1^) were chosen as the analytical band, whereas the peaks of the aromatic C..C bond str vibrations (1607 cm^−1^, not affected by the polymerization), were selected as the reference band. The DC% was then determined according to the equation: DC% = 100 × [1 − (ApC=C × AmC..C/AmC=C × ApC..C)], where A is the net peak absorbance height of set (p) and unset (m) materials at the specific wavenumbers.

### 2.2. Extent of Oxygen Inhibition

The extent of atmospheric oxygen inhibition on the free-radical polymerization of the adhesives was measured by the microscopic method. Briefly, five drops of each material were placed in wells and stored for 10 min (37 °C, dark/dry conditions) for solvent evaporation. Then an aliquot (~10 μL) was applied on a glass slide placed in a horizontal position on the reading table of a light transmission microscope (DM 4000B, Leica, Wetzlar, Germany) and covered by a transparent glass cover-slip with two spacers maintaining a constant sample thickness of 100 μm. Under these conditions resin-air contact was possible only between the slide and the slip, whereas the sample diameter (5–7 mm) ensured homogeneous light exposure. The materials were irradiated for 15 s with the LED curing unit, as before. The extent of the oxygen inhibited zone, oriented from the difference in the refractive index between set and unset materials, was photographed under standard magnification (200×) at three different areas, 10 min after light-curing and the mean value was used as representative of each specimen (n = 5/product). 

### 2.3. Hardness

Adhesive specimens (Ø = 7 mm, h = 5 mm, n = 8) were prepared by using molds of the same internal dimensions, placed and sealed on a glass plate. The adhesives were applied sequentially in 1 mm layers with a 10 min intermediate storage period (37 °C, dark/dry conditions, for solvent evaporation) and subsequent light-cured for 15 s per layer, as before. After application and solvent evaporation, the top layer was covered with a mylar strip attached to a glass-slide and light-cured. Each cylindrical specimen was stored at the same conditions for 24 h storage and then transferred to the reading table of a microhardness tester (HV 2000, Shimadzu, Tokyo, Japan) equipped with a Vickers indenter. Three indentations were performed on the directly irradiated surface of each specimen under 200 gf load, 10 s contact period and averaged. The indentations were made in a triangular mode, 1 mm distant to the specimen margins and the results were registered in Vickers micro-hardness (VHN). 

### 2.4. Interactions with Dentin

Dentin specimens were prepared from extracted sound third molars and kept in distilled water at 8 °C with the addition of 0.5 % sodium azide to inhibit bacterial growth. The crowns were horizontally sectioned at 1 mm distance below the occlusal dentin-enamel junction by using a low-speed hard tissue microtome (Isomet, Buhler, Lake Bluff, IL, USA). A second section, parallel to the first one, was performed at 1 mm distance apically and the occlusal surfaces of the dentin disks produced were polished with SiC papers up to 1000 grit-size in a grinding polishing machine (Dap-V, Struers, Ballerup, Denmark) at 200 rpm under water coolant, to create a standardized smear-layer. The reactivity with dentin and the dentin demineralization capacity of the adhesives, used as self-etch primers, were evaluated by reflection Fourier transform infrared microscopy (RFTIRM), employing an FTIR microscope (AutoImage, Perkin-Elmer, Buckinghamshire, UK) equipped with a liquid-N_2_ cooled mid-band mercury-cadmium telluride detector. A region was randomly selected at the center of the smear-layer covered specimens (n = 5/product) and the microscope stage coordinates were stored in the computer. Spectra were acquired under the following conditions: 2000–650 cm^−1^ wavenumber range, 4 cm^−1^ resolution, 400 × 300 μm^2^ aperture and 300 scans co-addition. The surfaces were then treated with each adhesive according to the manufacturers’ instructions. The adhesive films formed were not light-cured, stored for 15 min (37 °C, dark/dry conditions) and then rinsed off with water (5 mL) and acetone (3 mL). After air-drying (5 s), RFTIRM spectra were taken again at the same regions as before. All reflectance spectra were converted to absorbance by Kramers-Kroning transformation. The percentage extent of dentin demineralization (DM%) was evaluated on a relative basis by calculating the mineral (*v1,v3* –PO_4_ of Ca–P, 1185–885 cm^−1^) to the organic matrix (C=O str, amide I of collagen type I, ~1650 cm^−1^) peak area ratios after treatment (A) and normalizing these values by the corresponding values of native smear-layer covered surfaces before treatment (B) according to the equation: DM% = 100 × (1 − B/A). Transmission FTIRM spectra of uncured adhesive films applied on Ge windows were obtained as before (without rinsing) and used as reference for examination of the state of the adsorbed chemical groups of adhesive films on dentin, employing spectra subtraction techniques. Moreover, to identify the interaction of the phosphate monomers of the adhesives with dentin mineral, the complex –PO_4_ peak (1185–885 cm^−1^) was subjected to curve fitting, employing a Gaussian algorithm at standard width/variable shape mode and 2% zero baseline. The results of the area ratios of the new peaks appearing on treated dentin relative to the overall Ca-P peak expressed in percentage (AS%) were used to assess the contribution of these peaks. Peak fitting analysis was performed by PeakFit v.4.12 software (Seasolve, Framingham, MA, USA).

### 2.5. Shear Bond Strength with Dentin

Dentin specimens were prepared from extracted sound third molars kept stored as before. The tooth crowns were embedded in chemically-cured acrylic resin, horizontally sectioned at 1 mm distance below the occlusal dentin-enamel junction with the low-speed hard tissue microtome and polished with SiC papers (up to 1000 grit-size) in the grinding/polishing machine to create a standardized smear-layer. The specimens were then covered with an adhesive tape of 100 μm thickness that provided a hole of 3 mm in diameter, located at the center of dentin surfaces. For each adhesive, 10 specimens were randomly selected and subjected to the self-etch priming treatments according to the manufacturers’ information. The adhesive films were light-cured for 15 s with the LED curing unit. Molds of 3.5 mm internal diameter and 2 mm height were placed over the treated areas, filled with a resin composite (Tetric Evoceram, Ivoclar-Vivadent, Schaan, Liectenstein) in two 1 mm increments and light-cured for 20 s each. All the specimens were stored in water (1 week, 37 °C) and then subjected to shear loading at the dentin-composite interface with the notched-edge blade method [20] in a universal testing machine (Tensometer 10, Monsanto, Swidon, UK) operated at a crosshead speed of 0.5 mm/min. The debonded dentin surfaces were examined at 10× magnification under a stereomicroscope (M80, Leica, Wetzlar, Germany) to characterize the failure mode (Type I: Adhesive failure, Type II: Cohesive resin failure, Type III: Mixed type I and II failure and Type IV: Cohesive dentin failure).

### 2.6. Statistical Analysis

Since the DC%, extent of oxygen inhibition, VHN, DM% and AS% data passed normality and equal variance tests, one-way ANOVA and Tukey multiple comparison tests were used for the statistical analyses. The shear bond strength (SBS) data were analyzed by Weibull analysis. The shape or modulus parameter-*β* (defines the variability of the results, by expressing the size distribution of the flaws), the scale or B63.2 parameter-*σ*_0_, (defines the characteristic life, by indicating the strength value for which the 63.2% of the sample size were debonded) and the strength at 10% failure probability (*σ*_0.1_) of the Weibull distributions were calculated. Finally, a chi-square test was used to analyze the failure mode for each material. Pearson’s coefficient was used to investigate the correlation between the properties tested. The ANOVA, chi-square and correlation tests were performed by SigmaStat software (SigmaPlot v.12.5, Systat Software Inc, San Jose, CA, USA). For the Weibull analysis, the OriginLab software (v.9.1 SRO, Northampton, MA, USA) was used. For all cases, a 95% confidence level was selected (α = 0.05). 

## 3. Results

**Figure 1:**
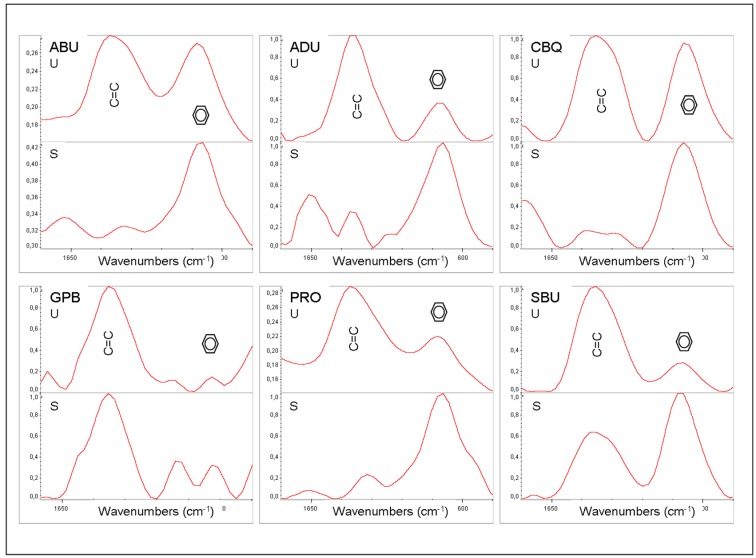
Attenuated total reflectance Fourier transform infrared (ATR-FTIR) spectra of the unset (U) and set (S) films of the universal adhesives used for degree of C=C conversion (DC) measurements, with the C=C and aromatic (hexagon) peak annotations (absorbance scale, 1700–1450 cm^−1^ wavenumber range).

**Figure 2:**
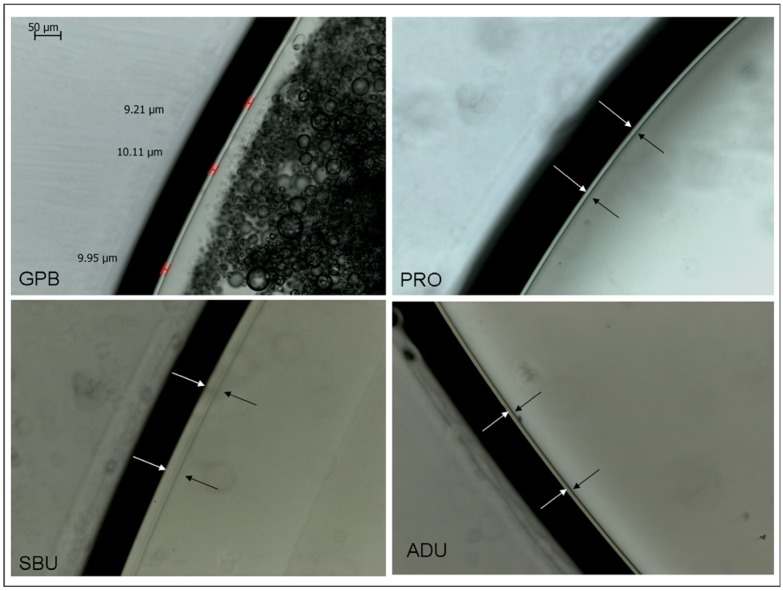
The extent of the O_2_-inhibition layer in representative universal adhesives. In G Premio Bond (GPB) image the measurement sites are marked. Arrows show the inhibited layer in Prelude One (PRO), Scotchbond Universal (SBU) and Adhese Universal (ADU). Note the pronounced phase separation in GPB (200× magnification, bar = 50 μm).

Representative spectra used for DC% calculation and microscopic images of the oxygen inhibition measurements are presented in Figure 1 and Figure 2.

The results of DC%, oxygen inhibition and VHN measurements are summarized in Table 2. High DC% values (77.7–81.5%) were registered for the adhesives, without statistically significant differences between them, except for GPB which exhibited the lowest values (67.2%). For the oxygen inhibition measurements, the ranking of the statistically significant differences in descending order was SBU > ADU, GPB, ABU > CBQ, PRO (*p* < 0.05). The microscopic images of GPB demonstrated excessive phase separation and bulk porosity, with the internal pore walls illustrating additional oxygen inhibited zones. The hardness values ranged between 1.1–6.6 VHN (10.8–64.7 MPa), with a statistical ranking of SBU > ADU > ABU > CBQ > PRO. It was not possible to record hardness measurements from GPB, since the material demonstrated a soft, gel consistency, contrary to all other materials which set under the specimen preparation conditions used.

**Figure 3:**
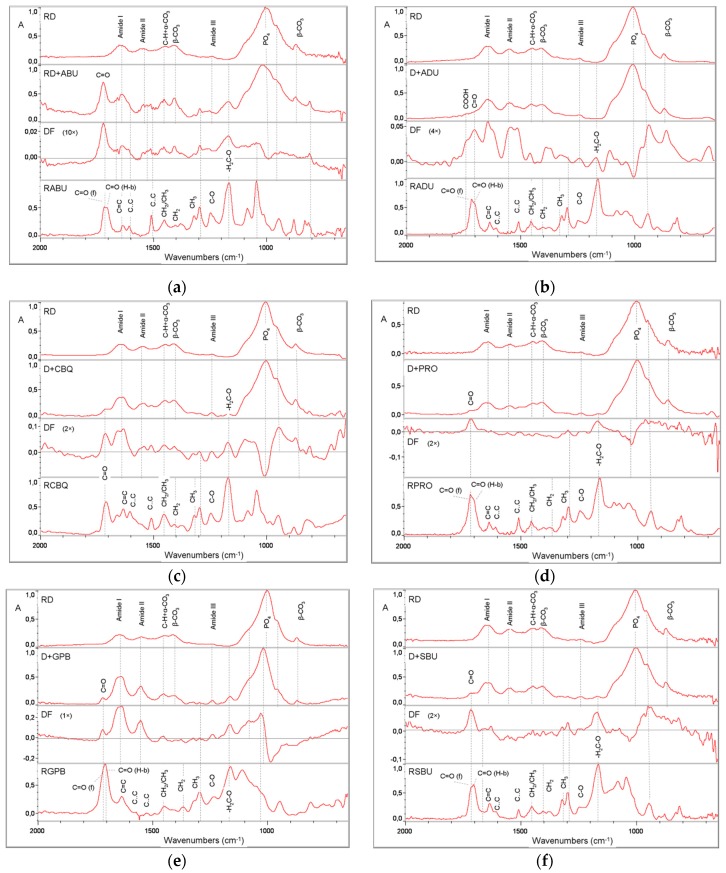
Representative reflection Fourier transform infrared microscopy (RFTIRM) spectra of reference dentin (RD), of dentin treated with the adhesives plus rinsing (RD + adhesive code), the difference spectra (DF, treated dentin-reference dentin) and the transmission spectra (RF+ adhesive code) of reference adhesives after solvent evaporation (absorbance scale, 2000–750 cm^−1^ wavenumber range). (**a**) ABU), (**b**) ADU, (**c**) CBQ, (**d**) GPB, (**e**) PRO, (**f**) SBU.

Figure 3a–f presents RFTIRM spectra (2000–650 cm^−1^ range) of smear-layer covered dentin specimens (RD), after adhesive treatments, storage and solvent rinsing (RD + adhesive code), the corresponding difference spectra (DF: treated minus native smear-layer covered dentin) and the transmission reference spectra of unset adhesives after solvent evaporation (R + adhesive code). The spectra of reference dentin demonstrated the characteristic peaks of C=O str of R-CONHR (amide I, 1660–1635 cm^−1^), N–H bending (b) plus C–N str of R–CONHR (amide II, 1580–1535 cm^−1^), C–H str and C–H b (1470–1450 cm^−1^) overlapping on α-CO_3_ (1453 cm^−1^), β-CO_3_ (1405 and 870 cm^−1^), C–N str plus N–H b (amide III, 1270–1245 cm^−1^), and the complex –PO_4_ peak of calcium phosphates (1185–885 cm^−1^) [21]. The reference spectra of the adhesives demonstrated the characteristic peaks of C=O (1720–1699 cm^−1^ dependent on the free or H–bonded status), C=C (1635, 945 cm^−1^) C..C (aromatics, 1605, 1590, 800–700 cm^−1^) CH_2_ and CH_3_ (1457–1370 cm^−1^), C–O of ester (1240 cm^−1^ ), C–O of CH_2_–O (1170 cm^−1^) and the complex contributions of the P=O, P–O, P–O–C, CH_2_–OH, C–O–C at the 1250–900 cm^−1^ band range [22].

Treatment with the universal adhesives in the self-etch mode and subsequent solvent rinsing revealed residual resin C=O peaks (1720–1715 cm^−1^) in all primed dentin specimens. Based on the scale of difference spectra (DF), the more intense C=O peaks were identified in GPB, CBQ and SBU corresponding to the vibrational mode of non H-bonded C=O or carboxylic acids (1730–1720 cm^−1^). In the DF spectra of ABU, ADH, CBQ and GPB the amide I peaks were positive and the *v1,v3* –PO_4_ peaks negative, whereas in SBU the amide I peak was at the baseline and the *v1,v3* –PO_4_ peaks negative. These findings indicate that the primed dentin surfaces were demineralized. The results of the extent of demineralization (DM%) are presented in Table 3. ABU, ADU, PRO and SBU established a statistically homogeneous group with the lowest DM (10.6–13.2%), CBQ showed higher DM (16%), statistically significant from ABU and PRO, while GPB demonstrated the highest value of all (69.3%). In all cases, the dentin surfaces treated with the adhesives were free of smear-layer. 

**Figure 4:**
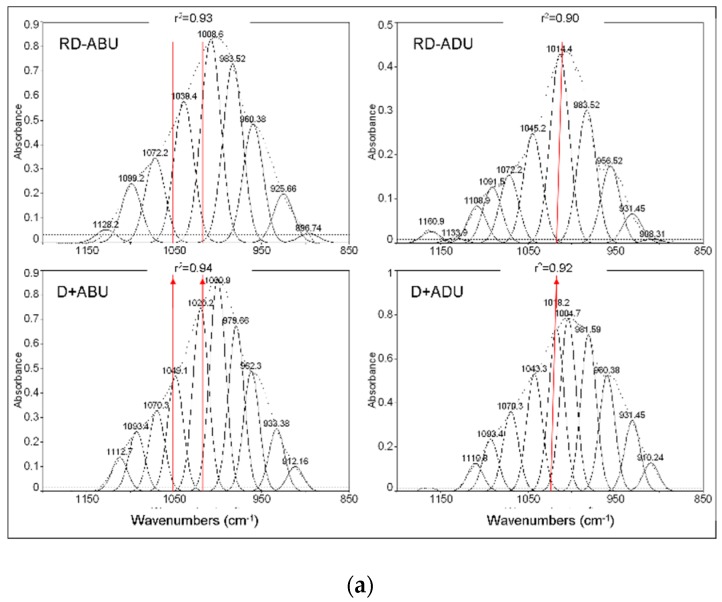

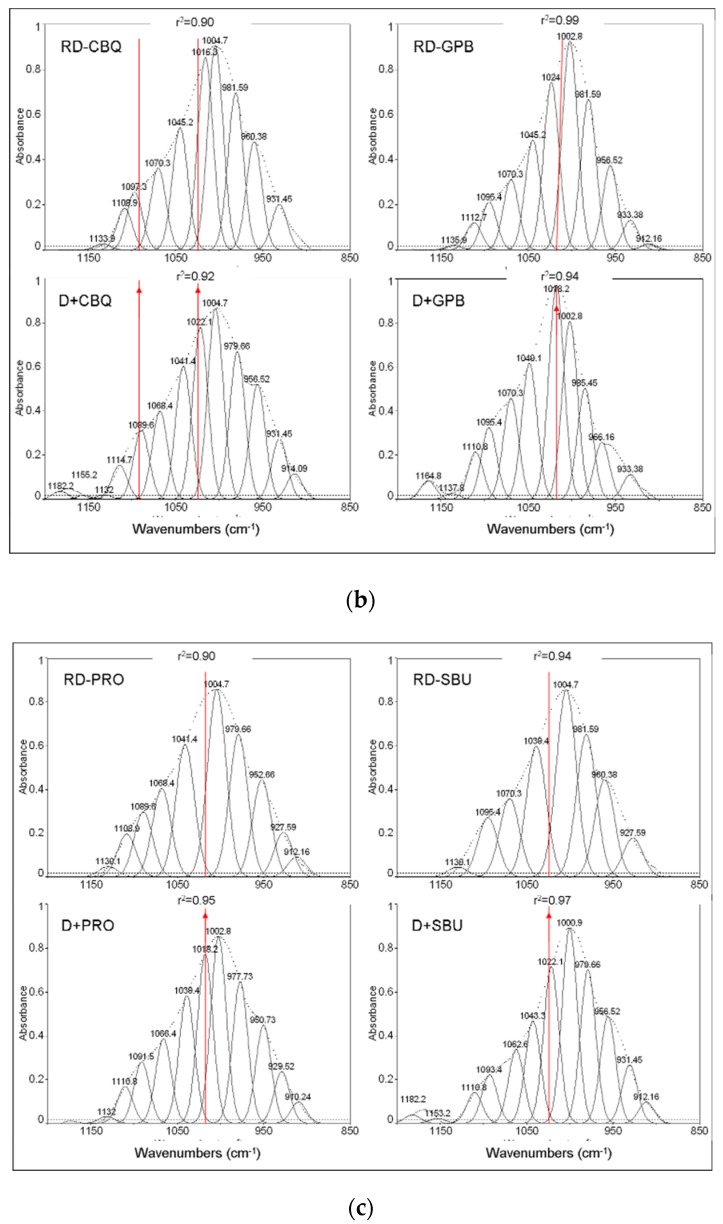
Gaussian curve-fitting of the complex calcium phosphate peak of reference dentin (RD) and of dentin treated with the adhesives after rinsing (RD + adhesive code) per product. The vertical lines denote the peaks appearing only on treated dentin, associated with calcium phosphates formed by the adhesive monomers (absorbance scale, 1185–885 cm^−1^ wavenumber range, r^2^: goodness of fit). (**a**) ABU and ADU, (**b**) CBQ and GPB, (**c**) PRO and SUB.

Representative graphs of the curve-fitted peaks of the *v1,v3* –PO_4_ domain (1185–885 cm^−1^) of dentin before and after treatment with the adhesives are illustrated in Figure 4. Treated and rinsed dentin surfaces demonstrated additional peaks at 1020 and 1049 cm^−1^ (ABU, GPB), 1022 and 1089 cm^−1^ (CBQ) and 1022 cm^−1^ (ADH, PRO, SBU) assigned to interaction of phosphate monomers with dentin mineral. The peak area ratios of these peaks relative to the overall Ca–P peak expressed in percentage (AS%) are summarized in Table 3. ABU and CBQ exhibited significantly higher values than GPB, PRO and SBU (*p* < 0.05).

**Figure 5:**
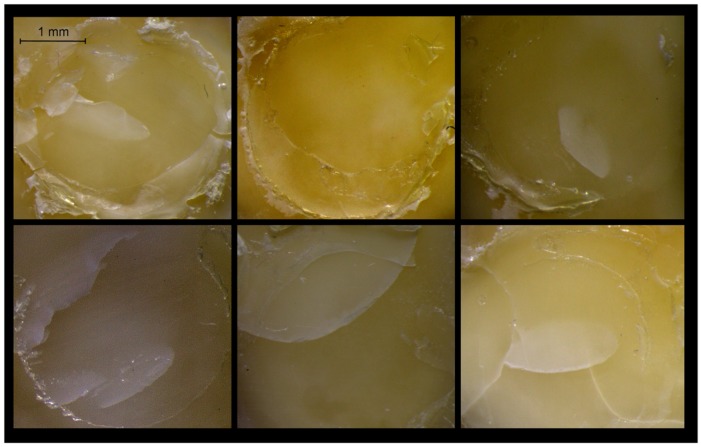
Representative stereomicroscopic images of dentin surfaces after debonding demonstrating type III failures (adhesive and resin cohesive) with various extent of dentin surface coverage (10× magnification, bar = 1 mm).

The results of the Weibull analysis (*β*, *σ*_0_, 95% C.I. for *σ*_0_, *σ*_0.1_ and 95% C.I. for *σ*_0.1_) are listed in Table 4. The ranking of the materials in reliability (*β*), from highest to lowest, was CBQ, SBU, PRO, ADU, GPB, ABU, with no statistically significant difference between them (*p* > 0.05). For the characteristic life (*σ*_0_), the ranking of the statistically significant differences was CBQ, ADU, ABU, SBU > PRO > GPB (*p* < 0.05), whereas for the 10% failure probability (*σ*_0.1_) SBU demonstrated the highest values with no statistically significant differences from CBQ, ADU, ABU, PRO and GPB the lowest, with no statistically significant differences from ADU, ABU, PRO. The *σ*_0.1_/*σ*_0_ ratios expressed in percentage were 67% (ABU, GPB), 68% (ADU), 74% (SBU, PRO) and 76% (CBQ). All failures were of type III (Figure 5), with a reduced amount of dentin surface covered by resin composite in the GPB debonded surfaces.

Pearson’s correlation coefficient analysis demonstrated statistically significant correlations only between nominal pH-DM% (r = −0.91, *p* = 0.012), DM%-*σ*_0_ (r = −0.84, *p* = 0.036) and DM%-DC% (r = −0.943, *p* = 0.005).

## 4. Discussion

Based on the results obtained in the present study, the null hypothesis has to be rejected, since significant differences were found between the materials tested in various properties. 

Universal dental adhesives were introduced as versatile multifunctional systems with reduced application steps, compatible with all dental hard tissue treatment modalities, capable of bonding to various restorative materials combined with appropriate surface treatments [10,23]. All the commercial products tested were based on the 10-MDP adhesive monomer with a documented bonding capacity with dentin [13], titanium, metal alloys [24,25] and polycrystalline ceramics [26,27,28]. In ADU and SBU, methacrylate-modified carboxylic acid copolymers are included, to facilitate dentin bonding under various degrees of hydration, whereas PRO contains a carboxyl methacrylate monomer. CBQ and SBU contain γ-methacryloxypropyl trimethoxysilane to mediate bonding with glass-ceramics. All adhesives, except GPB, are mild-pH, self-etch adhesives containing 2-HEMA with ethanol/water solvents, whereas GPB is a 2-HEMA-free, low-pH adhesive. All the adhesives, except GPB, contain the bulk BisGMA monomer, which provides strength and stiffness in the polymer network and hydrophobic aliphatic dimethacrylates for resilience and efficient cross-linking with the hydrophobic resin restoratives. In addition, ADU, CBQ and SBU contain colloidal silica film thickeners. 

Due to steric hindrance phenomena, dimethacrylate monomers do not polymerize fully and residual C=C bonds remain in the polymer network. DC depends on the chemical composition and polymerization conditions [29] and affects the mechanical properties [30,31], chemical stability and biological behavior of dental adhesive systems [32]. Although the universal adhesives tested contain high molecular weight dimethacrylates, such as BisGMA, with low conversion capacity, they exhibited high DC, since inclusion of low molecular weight monomethacrylates like 2-HEMA may lead to rapid copolymerization with the pendant C=C groups of the bulky monomers [33]. From the materials tested GPB demonstrated the lowest DC, possibly associated with the presence of many acidic monomers (10-MPD, 4-MET, MTDP) which interfere with conversion [34], the presence of residual solvents [35] and the absence of the non-acidic 2-HEMA monomer. It has been postulated that curing of acidic adhesives on dentin enhances DC in comparison with inert substrates due to acid neutralization and molecular orientation effects that create a more favorable condition for copolymerization [36]. A similar mechanism may apply also for bonding to enamel, since the presence of hydroxyapatite has been shown to improve the DC of self-etch adhesives [37]. The significant negative correlation between DM% and DC% documented in the present study may support this mechanism. Nevertheless, the DC monitored in the present study using an inert substrate (mylar strip) was within the range reported for a tooth substrate [16,38]. A possible explanation is the prolonged air-drying period (1 min, warm air), which may remove the solvents with high evaporation point (ethanol, water) more efficiently, minimizing thus their interference with the polymerization reaction [35]. 

Oxygen inhibition of free-radical polymerization, caused by the greater affinity of the radicals produced with atmospheric oxygen than themselves, results in the formation of an uncured surface layer, rich in stable peroxides. This is very important for thin film applications, such as sealants, adhesives and tooth desensitizers, since part or even the entire film may not set and subsequently be displaced from the treated surface or partially diffuse in the composite restorative paste; the latter may create incompatibility problems with slow setting materials (i.e. self-cure component of dual-cure materials) in the absence of an acid-compatible dual-cure activator [39,40]. The extent of oxygen inhibition in free-radical polymerization depends on the speed of the setting reaction; fast reactions, like light-curing, strongly reduce the time available for atmospheric oxygen to interact with the free-radical produced in comparison with slow setting self-cured materials. Therefore, the extent of inhibition is substantially reduced in the former [41]. Furthermore, high monomer viscosity, presence of fillers and incorporation of volatile solvents, create oxygen diffusion barriers contributing to reduced inhibition [42,43]. The microscopic method used for oxygen inhibition measurements has long been established in the field and is based on the differences in the refractive indices between set and unset regions [42]. The length of oxygen inhibition of most adhesives was ≤10 μm, values similar to several self-adhesive flowable liners/restoratives with much higher filler content [44] and almost half of the values reported for several unfilled sealants [41]. The presence of many low molecular weight and very reactive monomethacrylate monomers, such as 2-HEMA, and volatile solvents may explain the reduced sensitivity of these adhesives to environmental oxygen [45]. SBU demonstrated significantly higher inhibition, possibly attributed to differences in the setting rate dominated by the monomer and catalysis systems. Oxygen inhibition was not limited to the adhesive film margins exposed to air, but also was evident in adhesive films with entrapped air bubbles. The structural defects produced and the chemical degradation of the oxygen inhibited layer overtime, may impair the long-term stability of the adhesive layer [46]. Another important finding of the microscopic evaluation of the adhesive films was the phase separation identified in GPB, a typical observation in HEMA-free materials caused by the immiscibility of hydrophobic and amphiphilic monomers, upon solvent evaporation [47]. Such phenomena may create excessive differential monomer diffusion into demineralized dentin, especially for GPB, which has the lowest pH and therefore the strongest demineralization capacity.

Hardness of thin films, expressing their resistance to plastic deformation, is considered as an important parameter for bonding agents placed at the critical interface between dentin and restorative materials. Although nano- and indentation-hardness tests failed to predict the performance of dental adhesives subjected to conventional bond strength testing, a moderate correlation was established between Vickers indentation hardness and a scratch bond strength test with dentin [15]. In the present study, the VHN values were recorded on the surface of thick specimens under a low force to obtain an acceptable specimen thickness to indent depth ratio (≥10), excluding thus substrate interferences [48]. Preliminary experiments were performed for proper load selection to minimize any rebound effect. The universal adhesives tested demonstrated low VHN values. Since the adhesives were not water-stored and contain very reactive methacrylates, the low hardness values should be appended to a low cross-linking capacity and the plasticizing effect of residual solvents (water, ethanol, acetone). For GPB it was impossible to measure hardness, since the specimens had a soft gel consistency and poor cohesive strength, probably caused by increased solvent retention after storage. This solvent fraction may be associated with the lower fatigue strength and presence of voids and cracks documented for this product [49]. An interesting finding was the highest VHN of SBU and ADU, which both contain carboxylic acid polymers. These polymers may absorb water via hydrogen bonding, reducing thus the amount of the labile fraction, which may be implicated with side-reactions. As expected, the high DC values did not correlate with the VHN values, because high conversion of flexible monomers does not provide rigidity in the polymer network; the cross-linking density and the monomer structure seem to dominate the stiffness of the final product and the hardness values accordingly.

The strong negative correlation between the nominal pH values and the DM% implies that the predominant interaction of the universal adhesives with the dentin was demineralization. All of the treated dentin surfaces were free of a smear-layer, indicating that the adhesives were efficient in removing this fraction and demineralize subsurface dentin. The detection of residual ester (C=O) peaks (1720–1708 cm^−1^) on treated dentin surfaces after solvent rinsing (removal of water-soluble Ca–P salts by water and physisorbed resin by acetone) shows that some monomers were in a chemisorbed state. In most cases the difference spectra revealed the presence of freely vibrating (non H–bonded) ester bonds (~1720 cm^−1^ in ABU, CBQ, GPB, PRO) suggesting that the adsorbed monomer was attached to the substrate via the hydrophilic terminal group opposite to the methacrylate moiety. The peak at 1165 cm^−1^, assigned to the C–O group, although strong in all reference transmission spectra, it was considerably reduced in difference spectra possibly reflecting the adsorbed fraction of the adhesive monomers on dentin. In ADU the difference spectra demonstrated peaks at ~1740 cm^−1^, typical of COOH. No such groups were identified in PRO and SBU, which contain carboxyl groups as well, indicating that additional composition-related factors related, may control such reactions. The major contribution of 10-MDP to the adhesion mechanism was investigated after Gaussian peak fitting of the Ca–P complex peak. In two materials (ABU, CBQ), two peaks possibly assigned to Ca–P salt formation were identified, whereas in all other products, only one was traced. Such variations in 10-MDP reactivity can be caused from the purity of the raw material [50], the type of solvents [35,51], the presence of co-monomers with reaction capacity with 10-MDP [52] and the pH of the adhesive. For the latter, it has been postulated that a pH of 2.5–3 is the most appropriate to mediate chemical bonding with dentin, when adhesives are used in the self-etch mode [28,53]. Considering that the Ca–P salt formation induced by the phosphate monomers contributes to the overall Ca–P peak area (1185–885 cm^−1^), the fraction of dentin mineral reacted with the phosphate monomers ranged from 16–17% (ADU, GPB, PRO, SBU) up to 24–26% (CBQ, ABU). Alternatively, the extent of dentin demineralization, in the absence of chemical adhesion, would be expected to be higher at an equal percentage. The possibility that the peaks assigned to Ca–P salts could be contributions of the silane components (MPTMS) in SUB and CBQ spectra is minimal, since the major peak at 1020–1024 cm^-1^ appeared also in the silane-free adhesives. The highest salt formation in CBQ and ABU may anticipate differences in the reactivity of 10-MDP due to the presence of various co-monomers; in CBQ and ABU only two adhesive monomers exist (10-MDP and 2-HEMA), whereas in the remaining there are three (10-MDP, 2-HEMA, carboxylic acid copolymers/monomers for ADU, SBU/PRO and 10-MDP, 4-MET plus MTDP in GMP). These co-monomers may induce phosphate group derivatization, reducing the affinity of 10-MDP to dentin. Such reactions have already been documented between 10-MDP and the silane component of universal adhesives [54].

The reliability (*β*) of the adhesives in SBS testing showed no statistically significant differences, although the main values ranged from 5.1 to 9.7. This should be attributed to the wide range of confidence intervals found in CBQ and SBU. For the *σ*_0_ values the only statistically significant difference was found in GPB, which demonstrated the lowest values. This may be explained by the low pH of the adhesive, which may not offer the advantages of superficial demineralization and complete resin diffusion up to the demineralization front documented for mild self-etch adhesives [55]. Actually the strong negative correlation between DM% and *σ*_0_ found in the present study validates these claims for the materials tested. In addition, for this material the problem associated with the very low cohesive strength of the film may be encountered. Nevertheless, two of the adhesives with significantly higher *σ*_0_ strength than GPB (PRO, SBU), demonstrated values r^2^ < 0.9. The 10% failure probability was chosen as a more clinically relevant parameter than *σ*_0_ (63.2% probability) [56]. Actually the 5% level used in several studies, but in the present study values were registered only for >7% probability level. The 10% values ranged for all materials between 67–74% of the *σ*_0_, which are quite high percentages. Therefore for these materials the early failures seem to be close to the characteristic life. The failure mode analysis demonstrated a complex type III pattern in all specimens (*p* > 0.05), with most composite material located at opposite directions to the loading sites, as a result of the bending stresses developed at the region [57]. 

In the present study, the bond strength of the universal adhesives with dentin was evaluated only in the self-etch mode for two main reasons: first, this mode is not inferior to the etch-and-rinse one in respect of bond strength values [3]; and second, it provides more durable bonding after prolonged water ageing, because of degradation of the resin-infiltrated collagen in the later [58]. Despite the fact that all of the universal adhesives tested were based on the 10-MDP adhesive monomer, inclusion of different co-monomers (cross-linkers or adhesion promoters), catalysts and solvents led to great variations in the adhesive film properties, which affected their reactivity with dentin and subsequently their bond strength. Problems associated with phase separation affected fundamental properties like degree of conversion and hardness, with a negative impact on interfacial strength. Although clinical studies are required to establish the relevance of laboratory findings and claims, it is quite interesting that materials developed under the same concept and designed with the same major adhesive compound (10-MDP) demonstrated such a diversity in their *in vitro* properties.

## Figures and Tables

**Table 1:** The classification and composition of the universal adhesives tested.

Material	Classification	Composition *	Manufacturer
Adhese Universal (ADU)	Mild (pH = 2.8)	10-MDP, 2-HEMA, BisGMA, MCAP, D3MA, highly dispersed silica, ethanol, water, photoinitiators	Ivoclar-Vivadent, Schaan, Liechtenstein
All-Bond Universal (ABU)	Ultra-Mild (pH = 3.1)	10-MDP, 2-HEMA, BisGMA, ethanol, water, photoinitiator	Bisco, Schaumburg, IL, USA
Clearfil Universal Bond Quick (CBQ)	Mild (pH = 2.3)	10-MDP, 2-HEMA, BisGMA, hydrophilic amide methacrylate, MPTMS, NaF, colloidal silica, photoinitiators	Kuraray Noritake Dental, Tokyo, Japan
G-Premio Bond (GPB)	Low (pH = 1.5)	10-MDP, 4-MET, MTDP, methacrylic acid ester, silica, acetone, water, photoinitiators	GC Corp., Tokyo, Japan
Prelude One (PRO)	Mild(pH = 2.8)	10-MDP, Methacryloyloxyalkyl acid carboxylate, 2-HEMA, BisGMA, ethanol	Danville Materials, S. Ramon, CA, USA
Scotchbond Universal (SBU)	Mild (pH = 2.7)	10-MDP, 2-HEMA, BisGMA, DCDMA, MPTMS, VP-copolymer, fumed silica, ethanol, water, photoinitiators	3M ESPE, St. Paul, MN, USA

* According to the manufacturers’ information. 10-MDP:10-methacryloyloxydecyl dihydrogenphosphate, 2-HEMA: 2-hydroxyethyl methacrylate, BisGMA: Bisphenol-A glycidyl dimethacrylate, MCAP:Methacrylated carboxylic acid polymer, D3MA:Decandiol dimethacrylate, MPTMS: γ-methacryloxypropyl trimethoxysilane, 4-MET: 4-methacryloxyethyl trimellitic acid, MDTP: Methacryloyloxydecyl dihydrogen thiophosphate, DCDMA: Decamethylene dimethacrylate, VP-copolymer: Methacrylate-modified polyalkenoic acid copolymer.

**Table 2:** Results of DC%, VHN and O_2_ inhibition (mean values and standard deviations in parentheses). Same superscripts letters show mean values with no statistically significant differences between products per property (*p* > 0.05).

PRODUCT	DC%	O_2_ Inhibition (μm)	VHN
ABU	77.7 (2.9)^a^	8.6 (0.7)^a^	3.2 (0.1)^a^
ADU	78.7 (3.7)^a^	9.7 (0.3)^a^	5.3 (0.2)^b^
CBQ	82.0 (1.6)^a^	6.1 (0.6)^b^	1.7 (0.3)^c^
GPB	67.2 (3.8)^b^	8.9 (1.2)^a^	---
PRO	81.5 (3.9)^a^	5.6 (0.1)^b^	1.1 (0.1)^d^
SBU	81.5 (3.3)^a^	18.6 (0.8)^c^	6.6 (0.3)^e^

**Table 3:** The results of dentin dimineralization (DM%) and percentage of the peak area assigned to salt formation vs. the overall calcium phosphate peak (AS%, means values and standard deviation in parentheses). Same superscript letters show values with no statistically significant difference between the products per property (*p* > 0.05).

PRODUCT	DM%	AS%
ABU	12.9 (1.9) ^a^	26 (2.8) ^a^
ADU	12.5 (4.2) ^a,b^	16.8 (1.9) ^b^
CBQ	16.0 (1.4) ^b^	24 (3.6) ^a^
GPB	69.3 (4.9) ^c^	17.2 (2.6) ^b^
PRO	10.6 (3.1) ^a^	17.1 (2.1) ^b^
SBU	13.2 (2.9) ^a,b^	15.9 (1.4) ^b^

**Table 4:** The results of the Weibull analysis of the shear bond strength test. Same superscript letters show values with no statistically significant difference between the products per parameter (*p* > 0.05).

PRODUCT	Shape Parameter-*β* (95% CI)	Scale Parameter-*σ*_0_ (95% CI)/MPa	r^2^	10% Failure Probability-*σ*_0.1_ (95% CI)/MPa
ABU	5.1^a^ (3.4–8.9)	27.2^a^ (24.1–30.6)	r^2^ = 0.92	18.2^a,b^ (12.4–22.5)
ADU	6.2^a^ (3.9–10)	28.3^a^ (25.5–31.4)	r^2^ = 0.98	19.3^a,b^ (13.6–23.7)
CBQ	9.7^a^ (5.9–15.9)	29.3^a^ (27.4–31.3)	r^2^ = 1	22.3^a^ (17.2–26)
GPB	5.6^a^ (3.4–9)	16.6^b^ (14.7–18.7)	r^2^ = 0.92	11.1^b^ (7.8–14)
PRO	6.2^a^ (4–9.8)	22.5^c^ (20.2–25)	r^2^ = 0.84	16.7^a,b^ (12.5–19.9)
SBU	9.3^a^ (5.7–15.1)	27^a^ (25.2–29)	r^2^ = 0.82	19.9^a^ (15.4–24.2)

## References

[1] De Munck J., Van Landuyt K., Peumans M., Poitevin A., Lambrechts P., Braem M., Van Meerbeek B. (2005). A critical review of the durability of adhesion to tooth tissue: methods and results. J. Dent. Res..

[2] Salz U., Bock T. (2010). Testing adhesion of direct restoratives to dental hard tissue - a review. J. Adhes. Dent..

[3] Rosa W.L., Piva E., Silva A.F. (2015). Bond strength of universal adhesives: A systematic review and meta-analysis. J. Dent..

[4] Hanabusa M., Mine A., Kuboki T., Momoi Y., Van Ende A., Van Meerbeek B., De Munck J. (2012). Bonding effectiveness of a new ‘multi-mode’ adhesive to enamel and dentine. J. Dent..

[5] Perdigao J., Sezinando A., Monteiro P.C. (2012). Laboratory bonding ability of a multi-purpose dentin adhesive. Am. J. Dent..

[6] Chen C., Niu L.N., Xie H., Zhang Z.Y., Zhou L.Q., Jiao K., Chen J.H., Pashley D.H., Tay F.R. (2015). Bonding of universal adhesives to dentine--Old wine in new bottles?. J. Dent..

[7] Wagner A., Wendler M., Petschelt A., Belli R., Lohbauer U. (2014). Bonding performance of universal adhesives in different etching modes. J. Dent..

[8] Saikaew P., Chowdhury A.F., Fukuyama M., Kakuda S., Carvalho R.M., Sano H. (2016). The effect of dentine surface preparation and reduced application time of adhesive on bonding strength. J. Dent..

[9] Hirai K., Tsujimoto A., Nojiri K., Ueta H., Takamizawa T., Barkmeier W.W., Latta M.A., Miyazaki M. (2017). Influence of photoirradiation conditions on dentin bond durability and interfacial characteristics of universal adhesives. Dent. Mater. J..

[10] Nagarkar S., Theis-Mahon N., Perdigao J. (2019). Universal dental adhesives: Current status, laboratory testing, and clinical performance. J. Biomed. Mater. Res. B Appl. Biomater..

[11] Breschi L., Maravic T., Cunha S.R., Comba A., Cadenaro M., Tjaderhane L., Pashley D.H., Tay F.R., Mazzoni A. (2018). Dentin bonding systems: From dentin collagen structure to bond preservation and clinical applications. Dent. Mater..

[12] Wang R., Shi Y., Li T., Pan Y., Cui Y., Xia W. (2017). Adhesive interfacial characteristics and the related bonding performance of four self-etching adhesives with different functional monomers applied to dentin. J. Dent..

[13] Yoshida Y., Nagakane K., Fukuda R., Nakayama Y., Okazaki M., Shintani H., Inoue S., Tagawa Y., Suzuki K., De Munck J. (2004). Comparative study on adhesive performance of functional monomers. J. Dent. Res..

[14] Carrilho E., Cardoso M., Marques Ferreira M., Marto C.M., Paula A., Coelho A.S. (2019). 10-MDP Based Dental Adhesives: Adhesive Interface Characterization and Adhesive Stability-A Systematic Review. Materials.

[15] Kusakabe S., Rawls H.R., Hotta M. (2016). Relationship between thin-film bond strength as measured by a scratch test, and indentation hardness for bonding agents. Dent. Mater..

[16] Loguercio A.D., Munoz M.A., Luque-Martinez I., Hass V., Reis A., Perdigao J. (2015). Does active application of universal adhesives to enamel in self-etch mode improve their performance?. J. Dent..

[17] Cardenas A.M., Siqueira F., Rocha J., Szesz A.L., Anwar M., El-Askary F., Reis A., Loguercio A. (2016). Influence of Conditioning Time of Universal Adhesives on Adhesive Properties and Enamel-Etching Pattern. Oper. Dent..

[18] Jang J.H., Lee M.G., Woo S.U., Lee C.O., Yi J.K., Kim D.S. (2016). Comparative study of the dentin bond strength of a new universal adhesive. Dent. Mater. J..

[19] Rueggeberg F.A., Hashinger D.T., Fairhurst C.W. (1990). Calibration of FTIR conversion analysis of contemporary dental resin composites. Dent. Mater..

[20] ISO 29022:2013 (2013). Dentistry-Adhesion-Notched-Edge Shear Bond Strength Test.

[21] Verdelis K., Lukashova L., Wright J.T., Mendelsohn R., Peterson M.G., Doty S., Boskey A.L. (2007). Maturational changes in dentin mineral properties. Bone.

[22] Spencer P., Wang Y., Katz J.L., Misra A. (2005). Physicochemical interactions at the dentin/adhesive interface using FTIR chemical imaging. J. Biomed. Opt..

[23] Lumkemann N., Eichberger M., Stawarczyk B. (2019). Different surface modifications combined with universal adhesives: the impact on the bonding properties of zirconia to composite resin cement. Clin. Oral. Investig..

[24] Tsuchimoto Y., Yoshida Y., Mine A., Nakamura M., Nishiyama N., Van Meerbeek B., Suzuki K., Kuboki T. (2006). Effect of 4-MET- and 10-MDP-based primers on resin bonding to titanium. Dent. Mater. J..

[25] Ikemura K., Kojima K., Endo T., Kadoma Y. (2011). Effect of the combination of dithiooctanoate monomers and acidic adhesive monomers on adhesion to precious metals, precious metal alloys and non-precious metal alloys. Dent. Mater. J..

[26] Thompson J.Y., Stoner B.R., Piascik J.R., Smith R. (2011). Adhesion/cementation to zirconia and other non-silicate ceramics: where are we now?. Dent. Mater..

[27] Llerena-Icochea A.E., Costa R.M., Borges A., Bombonatti J., Furuse A.Y. (2017). Bonding Polycrystalline Zirconia With 10-MDP-containing Adhesives. Oper. Dent..

[28] Van Meerbeek B., Yoshihara K., Yoshida Y., Mine A., De Munck J., Van Landuyt K.L. (2011). State of the art of self-etch adhesives. Dent. Mater..

[29] Baroudi K., Saleh A.M., Silikas N., Watts D.C. (2007). Shrinkage behaviour of flowable resin-composites related to conversion and filler-fraction. J. Dent..

[30] Ferracane J.L., Greener E.H. (1986). The effect of resin formulation on the degree of conversion and mechanical properties of dental restorative resins. J. Biomed. Mater. Res..

[31] Pongprueksa P., De Munck J., Inokoshi M., Van Meerbeek B. (2018). Polymerization efficiency affects interfacial fracture toughness of adhesives. Dent. Mater..

[32] Santerre J.P., Shajii L., Leung B.W. (2001). Relation of dental composite formulations to their degradation and the release of hydrolyzed polymeric-resin-derived products. Crit. Rev. Oral. Biol. Med..

[33] Eliades G. (1994). Clinical relevance of the formulation and testing of dentine bonding systems. J. Dent..

[34] Hanabusa M., Yoshihara K., Yoshida Y., Okihara T., Yamamoto T., Momoi Y., Van Meerbeek B. (2016). Interference of functional monomers with polymerization efficiency of adhesives. Eur. J. Oral. Sci..

[35] Ikeda T., De Munck J., Shirai K., Hikita K., Inoue S., Sano H., Lambrechts P., Van Meerbeek B. (2005). Effect of evaporation of primer components on ultimate tensile strengths of primer-adhesive mixture. Dent. Mater..

[36] Gaintantzopoulou M., Rahiotis C., Eliades G. (2008). Molecular characterization of one-step self-etching adhesives placed on dentin and inert substrate. J. Adhes. Dent..

[37] Zhang Y., Wang Y. (2012). The effect of hydroxyapatite presence on the degree of conversion and polymerization rate in a model self-etching adhesive. Dent. Mater..

[38] Munoz M.A., Sezinando A., Luque-Martinez I., Szesz A.L., Reis A., Loguercio A.D., Bombarda N.H., Perdigao J. (2014). Influence of a hydrophobic resin coating on the bonding efficacy of three universal adhesives. J. Dent..

[39] Tay F.R., Pashley D.H., Yiu C.K., Sanares A.M., Wei S.H. (2003). Factors contributing to the incompatibility between simplified-step adhesives and chemically-cured or dual-cured composites. Part I. Single-step self-etching adhesive. J. Adhes. Dent..

[40] Suh B.I., Feng L., Pashley D.H., Tay F.R. (2003). Factors contributing to the incompatibility between simplified-step adhesives and chemically-cured or dual-cured composites. Part III. Effect of acidic resin monomers. J. Adhes. Dent..

[41] Lekka M.P., Papagiannoulis L., Eliades G.C., Caputo A.A. (1989). A comparative in vitro study of visible light-cured sealants. J. Oral. Rehabil..

[42] Ruyter I.E. (1981). Unpolymerized surface layers on sealants. Acta Odontol. Scand..

[43] Gauthier M.A., Stangel I., Ellis T.H., Zhu X.X. (2005). Oxygen inhibition in dental resins. J. Dent. Res..

[44] Eliades A., Birpou E., Eliades T., Eliades G. (2013). Self-adhesive restoratives as pit and fissure sealants: a comparative laboratory study. Dent. Mater..

[45] Pashley D.H., Tay F.R., Breschi L., Tjaderhane L., Carvalho R.M., Carrilho M., Tezvergil-Mutluay A. (2011). State of the art etch-and-rinse adhesives. Dent. Mater..

[46] Hashimoto M., Fujita S., Endo K., Ohno H. (2009). In vitro degradation of resin-dentin bonds with one-bottle self-etching adhesives. Eur. J. Oral. Sci..

[47] Van Landuyt K.L., De Munck J., Snauwaert J., Coutinho E., Poitevin A., Yoshida Y., Inoue S., Peumans M., Suzuki K., Lambrechts P. (2005). Monomer-solvent phase separation in one-step self-etch adhesives. J. Dent. Res..

[48] Matyunin M.M.A., Demidov A.N., Karimbekov M.A. (2016). Thin coatings and films hardness evaluation. IOP Conf. Ser. Mater. Sci. Eng..

[49] Tsujimoto A., Barkmeier W.W., Takamizawa T., Watanabe H., Johnson W.W., Latta M.A., Miyazaki M. (2017). Comparison between universal adhesives and two-step self-etch adhesives in terms of dentin bond fatigue durability in self-etch mode. Eur. J. Oral. Sci..

[50] Yoshihara K., Nagaoka N., Okihara T., Kuroboshi M., Hayakawa S., Maruo Y., Nishigawa G., De Munck J., Yoshida Y., Van Meerbeek B. (2015). Functional monomer impurity affects adhesive performance. Dent. Mater..

[51] Chen Y., Lu Z., Qian M., Zhang H., Chen C., Xie H., Tay F.R. (2017). Chemical affinity of 10-methacryloyloxydecyl dihydrogen phosphate to dental zirconia: Effects of molecular structure and solvents. Dent. Mater..

[52] Tian F., Zhou L., Zhang Z., Niu L., Zhang L., Chen C., Zhou J., Yang H., Wang X., Fu B. (2016). Paucity of Nanolayering in Resin-Dentin Interfaces of MDP-based Adhesives. J. Dent. Res..

[53] Yoshihara K., Yoshida Y., Hayakawa S., Nagaoka N., Torii Y., Osaka A., Suzuki K., Minagi S., Van Meerbeek B., Van Landuyt K.L. (2011). Self-etch monomer-calcium salt deposition on dentin. J. Dent. Res..

[54] Chen B., Lu Z., Meng H., Chen Y., Yang L., Zhang H., Xie H., Chen C. (2019). Effectiveness of pre-silanization in improving bond performance of universal adhesives or self-adhesive resin cements to silica-based ceramics: Chemical and in vitro evidences. Dent. Mater..

[55] Carvalho R.M., Chersoni S., Frankenberger R., Pashley D.H., Prati C., Tay F.R. (2005). A challenge to the conventional wisdom that simultaneous etching and resin infiltration always occurs in self-etch adhesives. Biomaterials.

[56] Lim K., Yap A.U., Agarwalla S.V., Tan K.B., Rosa V. (2016). Reliability, failure probability, and strength of resin-based materials for CAD/CAM restorations. J. Appl. Oral. Sci..

[57] DeHoff P.H., Anusavice K.J., Wang Z. (1995). Three-dimensional finite element analysis of the shear bond test. Dent. Mater..

[58] Zhang Z.Y., Tian F.C., Niu L.N., Ochala K., Chen C., Fu B.P., Wang X.Y., Pashley D.H., Tay F.R. (2016). Defying ageing: An expectation for dentine bonding with universal adhesives?. J. Dent..

